# Influence of Childhood Asthma and Allergies on Occupational Exposure in Early Adulthood: A Prospective Cohort Study

**DOI:** 10.3390/ijerph16122163

**Published:** 2019-06-19

**Authors:** Orianne Dumas, Nicole Le Moual, Adrian J. Lowe, Caroline J. Lodge, Jan-Paul Zock, Hans Kromhout, Bircan Erbas, Jennifer L. Perret, Shyamali C. Dharmage, Geza Benke, Michael J. Abramson

**Affiliations:** 1 INSERM, U1168, VIMA: Aging and Chronic Diseases, Epidemiological and Public Health Approaches, F-94807 Villejuif, France; orianne.dumas@inserm.fr (O.D.); nicole.lemoual@inserm.fr (N.L.M.); 2 Univ Versailles St-Quentin-en-Yvelines, UMR-S 1168, F-78180 Montigny le Bretonneux, France; 3 Allergy and Lung Health Unit, Melbourne School of Population and Global Health, University of Melbourne, Parkville VIC 3010, Australia; lowea@unimelb.edu.au (A.J.L.); clodge@unimelb.edu.au (C.J.L.); jennifer.perret@unimelb.edu.au (J.L.P.); 4 Murdoch Childrens Research Institute, Royal Children’s Hospital, Melbourne VIC 3052, Australia; 5 Barcelona Institute of Global Health (ISGlobal), 08003 Barcelona, Spain; janpaul.zock@isglobal.org; 6 Universitat Pompeu Fabra (UPF), 08002 Barcelona, Spain; 7 CIBER Epidemiología y Salud Pública (CIBERESP), 28029 Madrid, Spain; 8 Department of Environmental Epidemiology, Institute for Risk Assessment Sciences, Utrecht University, 3584CM Utrecht, The Netherlands; h.kromhout@uu.nl; 9 School of Psychology and Public Health, La Trobe University, Melbourne VIC 3086, Australia; bircan.erbas@gmail.com; 10 Department of Epidemiology & Preventive Medicine, School of Public Health & Preventive Medicine, Monash University, Melbourne VIC 3004, Australia; geza.benke@monash.edu (G.B.); michael.abramson@monash.edu (M.J.A.)

**Keywords:** asthma, allergies, occupational exposure, healthy worker hire effect, longitudinal study

## Abstract

We aimed to determine whether history of asthma/allergies in childhood was associated with avoidance of jobs with exposure to asthmagens in early adulthood. The Melbourne Atopic Cohort Study recruited 620 children at high risk of allergic diseases at birth (1990–1994). Asthma, hay fever and eczema were evaluated by questionnaires during childhood. A follow-up in early adulthood (mean age: 18 years) collected information on the current job. Occupational exposure to asthmagens/irritants was evaluated using a job-exposure matrix. The association between history of asthma/allergies in childhood and working in a job with exposure to asthmagens/irritants was evaluated by logistic regression, adjusted for age, sex and parental education. Among 363 participants followed-up until early adulthood, 17% worked in a job with exposure to asthmagens/irritants. History of asthma (35%) was not associated with working in an exposed job (adjusted OR: 1.16, 95% CI: 0.65–2.09). Subjects with history of hay fever (37%) and eczema (40%) were more likely to enter exposed jobs (significant for hay fever: 1.78, 1.00–3.17; but not eczema: 1.62, 0.91–2.87). In conclusion, young adults with history of allergies were more likely to enter exposed jobs, suggesting no avoidance of potentially hazardous exposures. Improved counselling against high risk jobs may be needed for young adults with these conditions.

## 1. Introduction

Around 15% of adult-onset asthma may be attributable to occupational exposures [1]. Occupational exposures may also cause worsening/exacerbations of pre-existing asthma (including childhood onset asthma) [2,3]. Work-exacerbated asthma is estimated to occur in 20–25% of adults with asthma [2,3]. Hundreds of substances have been identified to cause asthma at the workplace [4,5]. In an Australian study, 47% of men and 40% of women were exposed to asthmagen(s) at work [6].

Reduction of occupational exposures has been suggested to be an intervention with high potential for primary and secondary prevention of asthma [6,7], and the early career a relevant period for preventive measures [8,9]. Individuals with a history of allergic diseases in childhood may have increased risk of work-related asthma in adulthood, and are a relevant target group for specific interventions [8,9,10]. At the individual level, avoidance of jobs with hazardous occupational exposures in individuals with pre-existing asthma or allergies may be beneficial [8,9]. However, in epidemiological studies, this selection phenomenon—“healthy hire effect”—may cause bias, and obscure evidence for the role of new hazards. Few studies have examined the impacts of asthma/allergies in childhood on job choices in young adults, and contrasting results have been reported [11,12,13,14,15]. Using data from an Australian cohort of young adults followed-up since birth, we investigated whether a history of asthma/allergies in childhood was associated with avoidance of exposed jobs in early adulthood.

## 2. Materials and Methods

The Melbourne Atopic Cohort Study (MACS) is a prospective cohort of 620 children with a parent or older sibling with a history of allergic diseases, recruited at birth (1990–94), and followed-up until early adulthood [16]. The MACS was set-up to study the natural history of allergic disease and their risk factors [16,17,18]. In this context, history of asthma/allergies (ever) in childhood was evaluated by questionnaires at ages 6–7 and 12 years, based on parental reports of episode(s) of asthma, hay fever or eczema in the past 12 months. 

The 18-year follow-up survey collected information on current job (“if you are currently working, what is your current job?”), which was coded according to the International Standard Classification of Occupations 1988. Occupational exposures were evaluated using the updated version of the occupational asthma specific job-exposure matrix (OAsJEM) [4]. This method has been described in detail elsewhere. Briefly, the OAsJEM assessments are based on consensus from a working group of international experts, using a standardized procedure. OAsJEM classified exposures to 30 asthmagens/irritants, into three categories: “high” (high probability of exposure and moderate-to-high intensity), “medium” (low-to-moderate probability or low intensity) and “unexposed”. Medium or high exposure to at least one of the 30 asthmagens/irritants was considered in the analyses. The reference group included participants who did not work or worked in jobs without exposure. We examined the association of history of asthma/allergies in childhood with occupational exposures in early adulthood, using logistic regression adjusting for age, sex and parental education.

Analyses were conducted among participants with information on asthma/allergies during childhood (n = 499) who completed the 18-year follow-up survey (n = 369). After excluding six participants with imprecise job descriptions, the analytic sample included 363 participants (51% male). 

## 3. Results

In childhood, 35% of the participants had a history of asthma, 37% hay fever and 40% eczema. At follow-up in early adulthood, participants were on average 17.9 years old (standard deviation: 1.3, range: 15.0–21.4), 28% had current asthma and 39% reported a current job (including casual work and/or apprenticeship). Reporting a current job was not associated with history of allergic diseases. The most frequent jobs included shop salespeople/cashiers (28%), waiters (14%), sport-related jobs (10%; e.g., sport teachers, lifeguards, referees), cooks (8%) and bakers (6%). Sixty-three participants worked in a job with medium or high exposure (later referred to as “exposed jobs”) to asthmagens/irritants, which represented 17% of all participants and 45% of those currently working. The most frequent occupational exposures included: flour, foods, enzymes and indoor cleaning. Participants working in exposed jobs had less educated parents (62% had completed tertiary education) than those not working or not in exposed jobs (80%, *p* = 0.003), but no difference was observed regarding age or sex. 

In multivariable models, history of asthma was not associated with working in an exposed job (Table 1). Subjects with history of hay fever were more likely to enter exposed jobs (odds ratio = 1.78, 95% confidence interval: 1.00–3.17). A similar non-significant trend was suggested for subjects with history of eczema (1.62, 0.91–2.87). No significant interactions by sex or parental education were observed, although the association with eczema was more pronounced in females. No association was observed between working in specific jobs and history of asthma, hay fever or eczema. The observed associations were not driven by a single specific exposure or group of exposure (e.g., high molecular weight agents, low molecular weight agents, or irritants). A sensitivity analysis, using only participants who worked in unexposed jobs as reference group (n = 78), showed the findings were similar.

## 4. Discussion

We did not find evidence of avoidance of potentially hazardous exposures in young Australian adults with history of asthma or allergies. Unexpectedly, we found that participants with a history of hay fever were more likely to enter exposed jobs. To date, prospective studies examining the role of asthma/allergies in childhood in job choices in young adulthood were all conducted in Europe. While some reported an association of childhood allergic rhinitis [14] or asthma [12,13] with avoidance of potentially hazardous jobs, others found no such association for any allergic disease [11,15].

Several factors may explain these discrepancies. Low socioeconomic position/education has been reported as a factor leading to less health-based job selection [13,19]. Although MACS included participants with relatively highly educated parents and potential concern about allergies [16], it does not necessarily imply awareness of occupational risk factors, which is generally low [20]. Moreover, physicians’ training regarding job counselling in patients with asthma/allergies, which may vary by country, is likely to influence job choices in early adulthood. Whether the participants received career advice was unknown in the current study. A few studies reported that such advice was received by only ~10% of young adults with asthma [9,10,20], and this proportion might be lower for other allergic conditions. We did not collect information regarding the reasons influencing job choices. However, career choice is influenced by many factors, and individuals may not identify their health status as a primary reason for job choice or avoidance. As an example, in a study of German vocational trainees, only 8% of the subjects with current symptoms of asthma, allergic rhinitis or atopic dermatitis indicated that their preferred job choice had been influenced by their diseases [11]. Despite this low awareness or low report of the influence of health status on job choice at individual level, a health-related job selection phenomenon has been observed in several studies at a population level [12,13,19]. Finally, the jobs reported in this young cohort were most likely casual jobs, potentially temporary or precarious. Despite adjustment for parental education, potential residual confounding by socio-economic position might partly explain the unexpected finding that participants with a history of hay fever were more likely to enter exposed jobs, as suggested previously in a UK study [14].

In the MACS cohort, the definition of asthma and allergic diseases was based on questionnaires and not on actual doctor-diagnosis. However, most previous studies used similar questionnaires [11,12,13,14,15]. It is unlikely that differences in disease assessment explain the discrepancies between studies. Occupational exposures were evaluated using a job-exposure matrix, which may have resulted in loss of precision in the risk estimates. Nonetheless, to limit misclassification, the OAsJEM was recently updated based on the most recent expert knowledge in work-related asthma. In addition, the former version of this job-exposure matrix [21] has been used in many studies and populations in which associations with asthma outcomes were reported [4,22,23,24,25], supporting its relevance to evaluate exposure to agents potentially a risk for asthma.

The current analysis examined occupational exposures in a population of very young adults, an exposure window rarely investigated [9]. Thus, most reported jobs are likely not representative of ultimate career choices. Nonetheless, we found that these jobs frequently involved exposure to asthmagens/irritants, and could have adverse health consequences in the long-term [26].

## 5. Conclusions

Improved pre-employment counselling may be needed for young adults with asthma or allergies [8,9]. In epidemiological studies of work-related asthma, history of allergic diseases and socio-economic position in childhood should be addressed as potential confounders.

## Figures and Tables

**Table 1:** Association of history of asthma and allergic diseases in childhood with work in an exposed* job in early adulthood.

	Working in An Exposed* Job Adjusted OR (95% CI)			
	History of Asthma	History of Hay Fever	History of Eczema	Any of the 3 Conditions
**All ^†^**	1.16 (0.65–2.09)	1.78 (1.00–3.17)	1.62 (0.91–2.87)	1.51 (0.81–2.84)
**According to sex ^‡^**				
Men (n = 184)	1.38 (0.57–3.34)	1.87 (0.79–4.41)	0.86 (0.33–2.21)	1.59 (0.61–4.12)
Women (n = 179)	0.99 (0.44–2.22)	1.64 (0.75–3.57)	2.52 (1.16–5.47)	1.41 (0.61–1.27)
*p*-interaction	0.51	0.87	0.07	0.85
**According to parental education level ^§^**				
Low level (n = 85)	1.24 (0.43–3.56)	2.72 (0.97–7.59)	1.58 (0.56–4.46)	1.69 (0.53–5.41)
High level (n = 278)	1.11 (0.54–2.27)	1.41 (0.69–2.86)	1.60 (0.79–3.20)	1.37 (0.64–2.91)
*p*-interaction	0.72	0.27	0.85	0.73

OR, odds ratio; CI, confidence interval. * Medium or high level of exposure to asthmagens/irritants. Reference group: not working or working in a job without occupational exposure to asthmagens/irritants, according to the occupational asthma specific job-exposure matrix (OAsJEM) evaluation [3]. ^†^ Model adjusted for age at follow–up, sex, and parental education level. ^‡^ Model adjusted for age at follow–up and parental education level. ^§^ Model adjusted for age at follow–up and sex.
